# Dietary Supplements Use Among Individuals over 60 Years of Age in Poland

**DOI:** 10.3390/nu18071099

**Published:** 2026-03-30

**Authors:** Sonia Woch, Łukasz Wierucki, Krzysztof Flis, Małgorzata Sznitowska, Emilia Błeszyńska-Marunowska, Tomasz Zdrojewski, Piotr Bandosz

**Affiliations:** 1 Department of Preventive Medicine and Education, Medical University of Gdansk, 80-210 Gdansk, Poland; 2 Department of Pneumology, University Clinical Center, 80-952 Gdansk, Poland; 3 Department of Applied Pharmacy, Medical University of Gdansk, 80-210 Gdansk, Poland; 4 Department of Occupational, Metabolic and Internal Diseases, Medical University of Gdansk, 81-516 Gdynia, Poland; e.bleszynska@gumed.edu.pl

**Keywords:** aging, geriatrics, dietary supplements, food supplements

## Abstract

**Background/Objectives:** Dietary supplements (DSs) are widely available on the pharmaceutical market. Their consumption frequency has been observed to increase over the years, especially with age. To the best of our knowledge, this is the first study in Poland and Central-Eastern Europe to analyze the use of dietary supplements (DSs) based on a nationally representative, random sample of the elderly population. **Methods:** We examined 5987 individuals aged 60 years and older to assess the prevalence and types of DSs they use, with particular attention to socio-economic factors associated with their consumption. It also aimed to determine whose recommendations were followed when choosing supplements. **Results:** The percentage of individuals taking any DSs was 32.1% (95% CI: 29.8–34.4), with a higher proportion among women (38.7% (95% CI: 35.7–41.7)) compared to men (22.8% (95% CI: 20.6–25.1)). A multivariable analysis showed that DSs were more likely to be used by individuals with higher education (Prevalence Ratio (PR) 1.74 (95% CI: 1.47–2.06) for individuals with higher education vs. those with primary education). A link between age and DSs consumption was also observed, with the highest consumption rates found among those in their 80s (PR 1.46 (95% CI: 1.30–1.63) vs. the 60–69 age group). The most commonly used ingredients were vitamins, taken by 76.2% (95% CI: 73.6–78.9) of respondents, minerals (55.9% (95% CI: 52.8–59)), and plant-based preparations (45.0% (95% CI: 42–48)). Although DSs were most often used based on a doctor’s recommendation (58.5% (95% CI: 54.5–62.6)), over a third of patients initiated their use independently. **Conclusions:** This significant proportion of individuals taking dietary supplements in Poland, often without consultation with healthcare professionals, suggests that these products may be used in the absence of objective indications, which may be associated with adverse effects and potential drug–supplement interactions. This represents an important implication for clinicians, who should always extend the medical history to include information on DSs use. The findings also highlight the need to strengthen regulatory frameworks to ensure the safe use of these products and to optimally protect the health of older adults.

## 1. Introduction

Dietary supplements (DSs) are defined as food products intended to supplement a regular diet, a concentrated source of nutrients or other substances that have a physiological effect, consumed in a form that allows for dosing [1]. They are widely available on the market and contain, among others, vitamins, minerals, prebiotics, probiotics, plant-derived substances, amino acids, enzymes, essential fatty acids, and others. However, DSs do not replace a properly balanced diet.

Well-documented benefits of DSs are related to certain clinical situations (e.g., folic acid supplementation in the prevention of neural tube defects and fetal heart defects) and nutritional deficiencies. Also, the benefits of supplementing vitamins A, C, E, beta-carotene, calcium, magnesium, selenium, zinc, and most other substances found in DSs have not been conclusively proven [2,3,4,5]. Therefore, clinical recommendations from scientific societies on the prevention of cardiovascular diseases (CVDs) and malignant tumors do not include the widespread use of DSs in primary prevention, while emphasizing the importance of proper diet [6,7].

Regulatory systems for DSs vary significantly between countries. In Europe, including Poland, they are classified as food products. In contrast to medicinal products, their production and distribution are not subject to strict regulatory requirements [1,8,9]. In the United States, under the Dietary Supplement Health and Education Act (DSHEA), DSs are also not subject to mandatory pre-market evaluation [10]. A more restrictive approach is adopted in countries such as Canada, where a licensing system is required prior to market authorization, and Japan, which applies a structured system of functional foods with varying levels of scientific evidence required for health claims [11,12]. These differences highlight substantial global regulatory heterogeneity, which should be considered when interpreting international patterns of dietary supplement use. Knowledge of the actual prevalence of DSs use in the population is of crucial importance for potential regulatory changes aimed at improving consumer safety.

Recent years have witnessed an increased consumption of DSs in developed countries. In the literature, many studies on DSs use are available; however, there are limited analyses focusing on the use of these preparations among older adults. Globally, between 64% and 72% of elderly individuals in the U.S. and 39% to 77% in Europe report using DSs [13,14,15,16,17,18]. Previous evaluations of DSs consumption rates among senior adults in Poland have been based on studies in local or selected populations. These studies showed large discrepancies in the estimated DSs consumption frequency, ranging from 28% to 69% [19,20,21]. The DSs market is growing at a very rapid pace in Poland [14,22]. According to data from the Chief Sanitary Inspectorate from March 2026, over 75,000 products classified as dietary supplements have been registered in the notification system for first market [23]. This increase is driven not only by medical or pharmaceutical advice, but also by intensive advertising campaigns [24]. In 2015, more than 20% of commercials aired on popular Polish TV channels were dedicated to dietary supplements [25].

Individuals aged over 60 years represent a particularly important patient population. On the one hand, older adults are at increased risk of nutritional deficiencies due to economic, psychological, and physiological factors [26,27]. On the other hand, an issue may arise with the unjustified use of these products, which can contribute to polypharmacy, polyherbacy, adverse effects, and exceeding the reference values for certain nutrients [18,28].

Moreover, many advertised DSs are described as strengthening, improving bodily functions, or even possessing medicinal properties, which may contribute to older adults perceiving these products as a simple and rapid way to improve health [18,29]. In addition, DSs are produced in the same forms as oral medicinal products, such as tablets, capsules, syrups, and drops, which may lead to their misperception as medications.

However, there have been no studies assessing these rates in the general older adults population in Poland, and the most recent available data are from ten years ago. Furthermore, to the best of our knowledge, no similar analyses have been conducted in Central-Eastern European countries. The aim of this study was to fill this gap by assessing the consumption frequency and types of DSs among elderly in Poland. We hypothesize that DSs use in adults aged over 60 is associated with sociodemographic and socioeconomic factors, and that patterns of use may differ from those observed in other countries. This study analyzed the relationships between supplement use and these factors, and also aimed to determine whose recommendations were followed when selecting a particular dietary supplement.

## 2. Materials and Methods

### 2.1. Study Population and Sample

The analysis was conducted based on the PolSenior2 cross-sectional epidemiological study conducted between 2018 and 2019. The study group consisted of the population of Polish residents aged 60 years and older, not institutionalized. The study was conducted in a randomly selected sample from across the country, using a three-stage, complex sampling design, stratified by place of residence, age, and gender. The national PESEL registry was the sampling frame for selecting individual respondents. Contact was attempted with 10,635 respondents, with 5987 interviews completed, resulting in an efficiency rate of 56.3%. All respondents were included in the present analysis.

The sample was representative of the Polish population by place of residence. Additionally, the oldest age groups were overrepresented to increase statistical power for estimating parameters within this subgroup. Overrepresentation was corrected during the post-stratification phase using weighting. A detailed description of sampling procedure is provided in a separate publication [30].

### 2.2. Study Protocol

The study involved a total of three home visits, during which medical, social, and economic data were collected, including information on dietary supplement use. Interviews were conducted by appropriately trained nurses using standardized questionnaires that considered gender, age (grouped into 10-year intervals), marital status, the size of the place of residence, and education. Financial status was assessed based on patients’ declarations, by asking the following question: “Which of the following statements best describes the financial situation in your household?” Good financial situation was defined as selecting one of the following: “It is enough for everything without special saving,” “I live frugally, and it is enough for everything,” or “I live very frugally to save for larger purchases.” Poor financial situation was indicated by selecting any of the following: “The money is only enough for the cheapest food and clothing or only for the cheapest food, and not enough for clothing,” or “There is not enough money even for the cheapest food and clothing.”

### 2.3. Definition and Categorization of Dietary Supplements

During the interview, respondents were asked the following question: “In the past week, did you take any medications, vitamins, nutritional supplements, or dietary supplements?” If the answer was yes, respondents were asked to present the packaging of the supplements. The brand names of the products were then recorded in the questionnaire (under the supervision of a trained nurse). Respondents were also asked by whom a given product was recommended (closed-ended question with possible answers: doctor, pharmacist, self-administered by the patient, friends or family, advertisement).

Based on the collected data, the products were classified by a physician (SW). Products whose brand names were registered in the notification system for first market introduction by the Chief Sanitary Inspectorate were considered dietary supplements [23]. It is important to note that no products listed in the Official List of Medicinal Products Authorized for Marketing in Poland (including vitamins, minerals, and herbs registered as medicines) were included in this study [31].

Among respondents reporting DSs use, an analysis of the individual ingredients contained in the preparations was conducted. The categorization of DSs based on their content was done following the report of the working group of the European Commission, with six groups established: minerals, vitamins, plant-derived products, probiotics and prebiotics, essential fatty acids, amino acids, enzymes, and others [32]. Classification was done based on the brand names of the products listed in the questionnaire and their composition as provided in the Chief Sanitary Inspectorate’s register of products subject to first market introduction notification [23]. For foreign products, classification was based on the dietary supplement registry in the country of manufacture. Multi-component products were assigned to more than one group.

### 2.4. Statistical Analysis

The evaluated DSs consumption frequency was expressed as a point estimate and 95% confidence interval. The analysis was conducted within subgroups based on age, education, size of the place of residence, financial situation, and marital status. Additionally, the relationship between dietary supplement use and age was visually assessed in gender groups using local regression (loess).

Differences in consumption frequency for all DSs and their subgroups were also assessed in multivariable analysis using Poisson regression, with robust variance estimation, which is appropriate for modeling prevalence data. Results were presented as central values of the prevalence ratio (PR) and 95% confidence intervals. A PR greater than 1 indicates a higher likelihood of DSs use compared to the reference category. Independent variables included gender, age, education, place of residence, financial situation, and marital status.

The consumption frequency for specific DSs groups was also determined, and the percentage of patients who were recommended these supplements by a doctor, pharmacist, friends or family, or who used these products based on personal decision or advertising was calculated. The relationship for consulting the use of a DSs with a professional was also assessed in a multivariable model using Poisson regression, including the same predictors as for DSs consumption frequency.

Calculations were performed considering the complex sampling design. Overrepresentation of older individuals in the sample was corrected to match the age structure of the Polish population using post-stratification weights. Statistical analysis was performed using the R software with a survey package (R for windows, version 4.3.3) [33,34].

## 3. Results

### 3.1. Characteristics of the Study Group

The study included 5987 participants, with women and men accounting for 51.1% (58.2% after weighting) and 48.9% (41.8% after weighting), respectively. The average age of respondents was 75.0 years. The majority of respondents had secondary or high school education, whereas those with higher education were a minority. In terms of place of residence and marital status, the majority of respondents were rural and small town, married residents. The vast majority of the study population described their financial situation as good.

**Figure 1:**
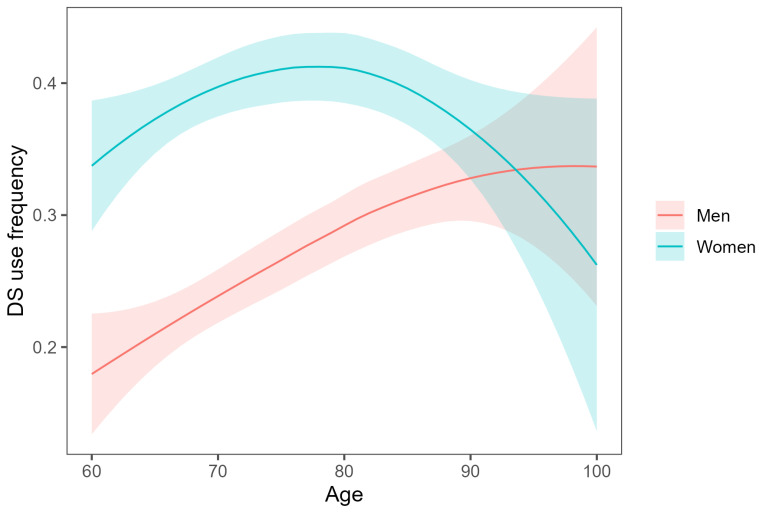
DSs use frequency among women, men and by age, including local regression (loess regression with parameters: polynomial degree = 2, α = 1). The shaded area represents the 95% confidence interval.

Table 1 presents detailed results for DSs consumption frequency (DSs) across various sociodemographic groups. For the entire analyzed age range, the percentage of older Poles reporting DSs use was 32.1% (95% CI: 29.8–34.4). In the early old age group, i.e., ≤80 years, the frequency of DSs consumption increased with age, with women using DSs nearly twice as often as men. Women aged 70–89 years were the most frequent DSs users, with over 40% of them reporting DSs consumption. The difference between the two gender groups diminished in successive decades of life. Among the oldest individuals (>90 years), the DSs consumption frequency was similar between genders, with approximately one-third of this subpopulation using such products. The relationship between DSs use and age is depicted graphically in Figure 1.

DSs were used more frequently by individuals with higher education. Respondents with higher or post-secondary education reported DSs use at a rate of 42.4% (95% CI: 36.7–48.1). This was declared by nearly half (48.6% (95% CI: 42.1–55.2)) of women in this group.

These products were also more commonly used by residents of large cities with populations exceeding 200,000 (37.7% (95% CI: 33.7–41.8)) compared to rural residents (29.8% (95% CI: 25.9–33.7)). However, no differences were observed among individuals living in other types of settlements.

There was no evidence to support the link between financial situation or marital status and DSs consumption frequency.

### 3.2. Recommendations for Dietary Supplement Use

Respondents most commonly reported following their physician’s recommendations (58.5% (95% CI: 54.5–62.6)). One-third of individuals reported their own independent decision to take dietary supplements (35.5% (95% CI: 31.5–39.4)). Opinions of pharmacists and friends or family played a significantly smaller role. This did not seem to depend on the seniors’ age. However, individuals aged >80 years, both women and men, were less likely to start using supplements independently compared to the youngest age group (60–69 years). Women were more likely than men to use dietary supplements based on a pharmacist’s recommendation (9.2% (95% CI: 6.4–11.9) vs. 4.0% (95% CI: 2.4–5.6)). No such relationship was observed for other sources of recommendations (Table 2).

It was found that women were more likely than men to consult a professional on the use of dietary supplements. Table 3 presents detailed results of a multivariate analysis conducted using Poisson regression, for those reporting the use of any dietary supplements. Declared use of dietary supplements based on advice from a doctor or pharmacist was a dependent variable.

### 3.3. Types of Dietary Supplements

The most commonly used DSs components were vitamins, taken by over three-quarters of respondents (76.2% (95% CI: 73.6–78.9)). Minerals were supplemented by more than half of seniors (55.9% (95% CI: 52.8–59)), followed by products containing plants and plant extracts (45.0% (95% CI: 42–48)). Nearly one-third of older individuals reported using products from the “other” category (30.0% (95% CI: 27.1–32.9)), while slightly more than one-fifth used fatty acids and phospholipids (21.3% (95% CI: 18.8–23.9)).

Amino acids (5.9% (95% CI: 4.5–7.3)) and probiotics or prebiotics (1.6% (95% CI: 0.9–2.4)) were least common. No cases of enzyme use were reported. No significant impact of age or gender on the frequency of use of specific supplement groups was observed. Detailed results are presented in Table 4.

Based on this analysis, it was confirmed that the frequency of DSs use was higher among women compared to men, both for any supplement used (PR = 1.69 (95% CI: 1.52–1.88)) and for the analyzed DSs groups. The highest DSs consumption frequency was observed in the 80–89-year age group (PR = 1.46 (95% CI: 1.30–1.63)) compared to the reference group of seniors aged 60–69 years. This was also true for vitamins (PR = 1.42 (95% CI: 1.22–1.65)) and minerals (PR = 1.70 (95% CI: 1.43–2.01)). The highest use of other DSs groups, such as essential fatty acids, amino acids, and others, was observed in individuals aged over 90 years. Regardless of the type of supplement, the frequency of DSs use increased with the level of education. For individuals with higher or post-secondary education, the prevalence ratio (PR) for any supplement use was 1.74 (95% CI: 1.47–2.06). However, other factors, such as financial situation, marital status, and place of residence, had no impact on DSs use. Detailed results of the multivariable analysis using Poisson regression are presented in Table 5.

## 4. Discussion

To the best of our knowledge, this is the first analysis in Poland (and Central and Eastern Europe) assessing the frequency of dietary supplement use based on a representative, randomized national sample of older adults among 60 years. Approximately one-third (32.1% (95% CI: 29.8–34.4)) of Polish seniors reported using DSs. Women used these products about 70% more frequently than men. The highest DSs consumptions was seen among individuals aged 80–89 years and those with higher education. Self-assessed financial situation, marital status, and place of residence were not associated with DSs consumption frequency.

The most commonly used DSs components were vitamins (76.2%), minerals (55.9%), plant-based products (45.0%), and essential fatty acids (21.3%). According to respondents, DSs use was most often based on a doctor’s recommendation (58.5%), although 43.6% of supplements were self-administered or chosen based on family or friends’ advice. In only 7.6% of cases, dietary supplementation was initiated based on pharmacist recommendations. Women were more likely to consult professionals about DSs usage than men.

### 4.1. Analysis of Key Findings

This study aligns with previous research demonstrating a correlation between older age and increased DSs consumption [13,35]. In Poland, DSs are used by 28% to 69% of seniors, depending on the study population and methodology [19,20,36,37,38,39]. Globally, between 64% and 72% of older adults in the U.S. and 39% to 77% in Europe report using DSs [13,14,15,16,17,18]. A possible explanation for the popularity of DSs among seniors may lie in their increased risk of nutritional deficiencies due to physiological changes, poor dietary habits, and the prevalence of chronic diseases [40]. Additionally, aggressive advertising targeting common health concerns among older adults is also a likely contributor [24,25].

It should be noted that the increasing prevalence of dietary supplement use in this age group is associated with the potential for adverse events. Recent evidence indicates that DSs use may be associated with clinically relevant drug–drug and drug–supplement interactions, particularly involving warfarin. In addition, excessive supplementation has been linked to adverse outcomes in older adults such as hypercalcemia, neuropathy, and nephropathy. Some DSs may also interfere with the interpretation of laboratory test results; for example, biotin supplementation may affect thyroid function assays, potentially leading to misinterpretation of diagnostic results [41].

Women consistently show higher DSs consumption than men, as seen in numerous studies, including this one [18,38,39,42,43,44]. Their increased interest in health and preventive care likely contributes to this trend [45]. For example, studies have noted higher vitamin D_3_ and calcium intake among women, probably linked to osteoporosis prevention [44,46].

Higher educational attainment has also been identified as an important factor influencing DSs use [42,47,48]. Both Polish and German studies have shown that individuals with higher education levels are the most frequent DSs users [38]. This finding aligns with the PolSenior2 study, which highlights education as a potential driver of health-conscious behavior. Given the above, the question arises whether the desire to enrich the basic diet with dietary supplements is justified and whether data on the actual efficacy of these products is readily available and presented in a way that is accessible to consumers.

It remains unclear whether economic status is a determinant of DSs consumption. While some studies indicate a positive association between income and DSs use, others, including this analysis, do not support such a link [38,49,50]. Substantial financial resources might enable access to DSs, but these findings suggest further investigation into dietary adequacy and health status across different income groups.

Marital status did not appear to influence DSs consumption in this study, which is consistent with findings from Germany and Taiwan [48,51]. However, some other research suggests that living alone may increase DSs usage, while married individuals or widows may be more likely to use DSs than divorced or single individuals [52].

Urban residency was associated with higher DSs consumption in univariate analysis, but not in multivariate analysis. Previous studies have noted a similar trend, suggesting that this relationship may be mediated by other factors, such as education [38].

### 4.2. Comparison with International Findings

This study has confirmed the international trends in DSs composition, with vitamins, minerals, and plant-based products being the most commonly used categories [43]. Similar patterns were observed in Japan, Germany, and the U.S., where vitamins and minerals dominate, followed by plant-based supplements [53]. Differences in DSs categorization across studies may contribute to variability in reported prevalence [43,54].

Doctors were the most frequently cited source of DSs recommendations, which is consistent with other analyses [36,37]. However, the high rate of self-administered DSs use and low reliance on pharmacists raise concerns.

### 4.3. Limitations and Strengths

The strengths of the PolSenior2 study include its national representativeness and rigorous data collection, verified by trained nurses who reviewed respondents’ actual DSs packaging. However, some limitations must be considered. Misunderstanding the definition of DSs and excluding some food products like teas may have led to underestimation. Furthermore, no information was collected regarding the declared reasons for DSs use or whether supplements were purchased as a substitute for over-the-counter (OTC) medications. The relatively short observation period (i.e., the last two weeks) may have excluded preparations used earlier or on an occasional basis. In addition, self-reported potential adverse effects following the initiation of a given supplement were not verified.

The present study constitutes a starting point and a basis for further analyses aimed at exploring the relationship between DSs use in this age group and factors such as physical activity, diet quality, and overall lifestyle. We also plan to extend the analysis to include the coexistence of chronic conditions, such as depression, diabetes, obesity, and cardiovascular diseases, as well as a history of hospitalizations.

## 5. Conclusions

The high prevalence of DSs use among older adults, often without professional consultation, suggests a potential risk of unnecessary adverse effects or harmful interactions. This is particularly concerning for seniors with limited financial resources, where purchasing DSs may reduce the budget for healthier food options. Older adults are vulnerable to marketing tactics that may misrepresent DSs benefits.

The present study underscores the need for more stringent pre-market regulations for dietary supplements, as well as improvements in post-market safety surveillance systems both in Poland and across Europe. Additionally, targeted education on balanced nutrition and evidence-based indications for dietary supplement use is essential to ensure optimal protection of older adults.

Pharmacists can play a critical role in guiding informed DSs choices and suggesting evidence-based alternatives when necessary. Clinicians should routinely assess DSs use during consultations to identify and address unnecessary supplementation. Women, individuals with higher education, and urban residents warrant particular attention due to their higher likelihood of DSs use.

## Figures and Tables

**Table 1:** The percentage of older adults reporting dietary supplement use by gender, age category, education level, place of residence, financial situation, and marital status. The 95% confidence interval (95% CI) is provided in parentheses.

Variable	Category	Men (%)	Women (%)	Total (%)
Age group	60–69 years	19.0 (16.0–22.1)	37.2 (33.0–41.4)	28.9 (25.9–31.9)
	70–79 years	26.6 (22.6–30.5)	40.8 (36.4–45.2)	35.1 (31.7–38.4)
	80–89 years	32.3 (27.6–37.1)	40.6 (36.7–44.6)	38.0 (34.9–41.0)
	90 and more years	34.9 (28.3–41.5)	33.0 (26.0–40.0)	33.4 (27.8–39.1)
Education	Primary/incomplete primary school	18.3 (13.9–22.6)	31.2 (26.3–36.0)	26.9 (23.2–30.5)
	Secondary school/vocational	22.0 (19.2–24.8)	38.6 (35.0–42.3)	30.9 (28.3–33.5)
	Higher and post-secondary	32.5 (26.0–39.0)	48.6 (42.1–55.2)	42. 4 (36.7–48.1)
Place of Residence	Rural	20.4 (16.7–24.2)	36.8 (31.5–42.0)	29.8 (25.9–33.7)
	City ≤ 200,000 inhabitants	23.1 (19.3–26.9)	36.7 (32.5–40.9)	30.9 (27.6–34.3)
	City > 200,000 Inhabitants	26.7 (23.5–29.9)	44.9 (39.6–50.3)	37.7 (33.7–41.8)
Financial Situation	Poor	25.9 (18.2–33.5)	36.9 (29.2–44.7)	33.0 (27.2–38.8)
	Good	22.8 (20.5–25.1)	38.9 (35.7–42.2)	32.1 (29.6–34.5)
Marital Status	Single	19.0 (16.0–22.1)	37.2 (33.0–41.4)	28.9 (25.9–31.9)
	Married	26.6 (22.6–30.5)	40.8 (36.4–45.2)	35.1 (31.7–38.4)
	Widowed	32.3 (27.6–37.1)	40.6 (36.7–44.6)	38.0 (34.9–41.0)
	Divorced	34.9 (28.3–41.5)	33.0 (26.0–40.0)	33.4 (27.8–39.1)

**Table 2:** DSs consumption frequency among individuals aged over 60 years by declared source of recommendations. The 95% confidence interval (95% CI) is provided in parentheses.

Source of Recommendation	Men (%)	Women (%)	Total (%)
Physician	56.4 (50.5–62.4)	59.4 (55.2–63.6)	58.5 (54.5–62.6)
Pharmacist	4.0 (2.4–5.6)	9.2 (6.4–11.9)	7.6 (5.5–9.7)
Independent decision	38.8 (33.2–44.3)	34.1 (29.8–38.4)	35.5 (31.5–39.4)
Friends or Family	5.6 (3.6–7.6)	5.6 (3.9–7.4)	5.6 (4.3–6.9)
Advertisement	1.4 (0.6–2.2)	3.0 (1.2–4.9)	2.5 (1.2–3.8)

**Table 3:** Factors associated with more frequent consultation on the use of a dietary supplement with a doctor or pharmacist, presented as prevalence ratios (PR). The 95% confidence interval (95% CI) is provided in parentheses. Ref.—reference group.

Variable	Category	Any Dietary Supplement PR (95% CI)
Gender	Men	Ref.
	Women	1.17 (1.03–1.32)
Age group	60–69 years	Ref.
	70–79 years	1.14 (1.03–1.26)
	80–89 years	1.20 (1.07–1.36)
	90+ years	1.29 (1.08–1.54)
Education	Primary or incomplete primary	Ref.
	Secondary, high school or vocational	0.91 (0.83–1.00)
	Higher or post-secondary	1.03 (0.90–1.18)
Place of Residence	Rural	Ref.
	City ≤ 200,000 inhabitants	0.99 (0.87–1.12)
	City > 200,000 inhabitants	1.04 (0.89–1.22)
Financial Status	Poor	Ref.
	Good	0.99 (0.80–1.22)
Marital Status	Married	Ref.
	Single	1.12 (0.95–1.31)
	Widowed	0.88 (0.77–1.00)
	Divorced	0.88 (0.69–1.12)

**Table 4:** Consumption frequency for specific groups of dietary supplements among individuals who used at least one dietary supplement. The 95% confidence interval (95% CI) is provided in parentheses.

Supplement Group	Men (%)	Women (%)	Total (%)
Vitamins	74.8 (70.1–79.4)	76.9 (73.7–80.0)	76.2 (73.6–78.9)
Minerals	55.2 (50.0–60.4)	56.2 (52.5–59.9)	55.9 (52.8–59.0)
Amino Acids	6.6 (3.8–9.4)	5.6 (4.1–7.2)	5.9 (4.5–7.3)
Probiotics/Prebiotics	2.7 (0.8–4.5)	1.2 (0.4–2.0)	1.6 (0.9–2.4)
Essential Fatty Acids	23.0 (19.1–27.0)	20.6 (17.4–23.8)	21.3 (18.8–23.9)
Plants or Plant Extracts	46.4 (40.5–52.2)	44.4 (41.1–47.7)	45.0 (42.0–48.0)
Other	32.3 (26.9–37.7)	29.0 (25.9–32.2)	30.0 (27.1–32.9)
Enzymes	0.0 (0.0–0.0)	0.0 (0.0–0.0)	0.0 (0.0–0.0)

**Table 5:** Prevalence ratio (PR) for the relationship between dietary supplement use and gender, age category, education level, place of residence, financial situation, and marital status. The 95% confidence interval (95% CI) is given in parentheses. Ref.—reference group; EFA—Essential Fatty Acids.

Variable	Category	Any Dietary Supplement PR (95% CI)	Vitamins PR (95%CI)	Minerals PR (95% CI)
Gender	Men	Ref.	Ref.	Ref.
	Women	1.69 (1.52–1.88)	1.78 (1.56–2.03)	1.72 (1.44–2.07)
Age Range	60–69 years	Ref.	Ref.	Ref.
	70–79 years	1.25 (1.11–1.41)	1.18 (1.03–1.34)	1.36 (1.15–1.59)
	80–89 years	1.46 (1.30–1.63)	1.42 (1.22–1.65)	1.70 (1.43–2.01)
	90+ years	1.34 (1.10–1.64)	1.36 (1.04–1.77)	1.52 (1.11–2.06)
Education	Primary or incomplete primary	Ref.	Ref.	Ref.
	Secondary, high school/vocational	1.35 (1.17–1.57)	1.48 (1.18–1.88)	1.52 (1.21–1.90)
	Higher or post-secondary	1.74 (1.47–2.06)	2.00 (1.58–2.53)	1.90 (1.39–2.60)
Place of Residence	Rural	Ref.	Ref.	Ref.
	City ≤ 200,000 inhabitants	0.94 (0.80–1.11)	0.90 (0.74–1.10)	0.82 (0.66–1.02)
	City > 200,000 inhabitants	1.10 (0.92–1.32)	1.12 (0.91–1.36)	1.05 (0.83–1.31)
Financial Situation	Poor	Ref.	Ref.	Ref.
	Good	0.93 (0.78–1.11)	0.96 (0.76–1.21)	1.01 (0.72–1.42)
Marital Status	Married	Ref.	Ref.	Ref.
	Single	1.02 (0.78–1.34)	1.08 (0.78–1.51)	1.17 (0.82–1.69)
	Widowed	0.93 (0.82–1.06)	0.91 (0.80–1.05)	0.90 (0.75–1.08)
	Divorced	1.04 (0.86–1.26)	1.10 (0.82–1.46)	1.26 (0.97–1.64)
**Variable**	**Category**	**EFA PR (95%CI)**	**Plants and Plant** **Extracts PR (95% CI)**	**Other PR** **(95% CI)**
Gender	Men	Ref.	Ref.	Ref.
	Women	1.54 (1.16–2.04)	1.55 (1.28–1.88)	1.38 (1.12–1.71)
Age range	60–69 years	Ref.	Ref.	Ref.
	70–79 years	1.43 (1.06–1.95)	1.30 (1.09–1.55)	1.29 (1.01–1.66)
	80–89 years	2.09 (1.48–2.95)	1.45 (1.21–1.73)	1.71 (1.38–2.12)
	90+ years	2.63 (1.54–4.49)	1.47 (1.02–2.12)	2.38 (1.64–3.45)
Education	Primary or incomplete primary	Ref.	Ref.	Ref.
	Secondary, high school/vocational	1.82 (1.25–2.63)	1.62 (1.28–2.05)	1.44 (1.07–1.94)
	Higher or post-secondary	2.74 (1.83–4.09)	2.12 (1.63–2.76)	2.34 (1.65–3.33)
Place of Residence	Rural	Ref.	Ref.	Ref.
	City ≤ 200,000 inhabitants	0.90 (0.65–1.25)	0.89 (0.69–1.14)	0.94 (0.71–1.25)
	City > 200,000 inhabitants	0.80 (0.58–1.10)	1.08 (0.79–1.47)	1.22 (0.86–1.73)
Financial Status	Poor	Ref.	Ref.	Ref.
	Good	1.02 (0.57–1.81)	0.97 (0.68–1.39)	0.87 (0.57–1.33)
Marital Status	Married	Ref.	Ref.	Ref.
	Single	1.58 (0.80–3.12)	1.16 (0.59–2.3)	1.18 (0.64–2.15)
	Widowed	0.89 (0.66–1.18)	1.06 (0.87–1.29)	0.84 (0.69–1.03)
	Divorced	1.05 (0.65–1.70)	1.25 (0.90–1.75)	1.26 (0.83–1.93)

## Data Availability

The original contributions presented in this study are included in the article. Further inquiries can be directed to the corresponding author.
